# *Eucalyptus* transgenic plants: from genetic transformation protocols to biosafety analysis

**DOI:** 10.1186/1753-6561-5-S7-P179

**Published:** 2011-09-13

**Authors:** Rochele Kirch, Leandro V Astarita, Eliane R Santarém, Giancarlo Pasquali

**Affiliations:** 1 Biotechnology Center, Federal University of Rio Grande do Sul, Porto Alegre, RS, Brazil; 2 Pontifical Catholic University of Rio Grande do Sul, Porto Alegre, RS, Brazil

## 

*Eucalyptus* is an exotic plant in Brazil, being originally from Australia. There are about 700 *Eucalyptus* species described and over 3,000 hybrids. It is one of the most planted tree genus in the world. The great economic interest on *Eucalyptus* trees is due to their fast growth, high productivity, great adaptability to different types of soils and climates, and also to the high versatility of their wood. *Eucalyptus* timber has applications for many different purposes such as cellulose pulp and paper production, electric poles, energy, charcoal, lumber and furniture. Given the economic importance of *Eucalyptus* in Brazil, it is of great interest to generate trees with superior characteristics that may result in considerable gains for the sector, particularly with regard to productivity and wood quality. One main goal of the present work is the definition of an efficient protocol for the genetic transformation and regeneration of transgenic *Eucalyptus* trees. We have so far obtained callus derived from leaves of *E.globulus* showing high capacity of *in vitro* regeneration. These calli were transformed with *Agrobacteriumtumefaciens* LBA4404 harboring the binary plasmid pGfpKan, containing the *green fluorescent protein* (*gfp*) gene as reporter. Regenerated plants were transferred to culture pots with MS medium. Leaves derived from each regenerated plant were collected and analyzed using confocal microscopy to investigate the presence of fluorescence, indicating successful transformation. Molecular assays are also being performed to confirm the independence of transformation events via the pattern of transgene integration into plant genomes. The commercial release of GMOs and its derivatives in Brazil is regulated by the National Technical Biosafety Commission (CTNBio) in terms of the Regulatory Resolution No. 5, March 12, 2008. A series of experiments must be performed in order to prove the equivalence between GM and non-GM plants concerning the effects on human and animal health as well as on the environment. We are conducting the evaluation of global gene expression among different lines of transgenic and non-GM *Eucalyptus* adult plants, already available in test-fields belonging to FuturaGene in Brazil. Leaf and stem samples of GM and controls were collected and stored at -80 °C. Total RNA and proteins from leaves and stems were extracted and quantified. Messenger RNAs are under sequencing in Illumina platforms, according to the mRNA-Seq protocol (Fasteris S.A.) Our idea is to compare transcript profiles among GM and non-GM tree samples, checking for possible pleiotropic effects of the transgenes. Protein profiles of the same individuals will also be analyzed to verify if the presence of the transgene influences the expression of other proteins in *Eucalyptus*. In order to do so, total proteins were extracted from samples and fractionated by 1D SDS-PAGE, cut off from gels and processed for posterior Mass Spectrometry sequencing.

**Financial Support:** MAPA/CNPq & FuturaGene.

